# Inflammation-based assessment for the risk stratification of mortality in patients with heart failure

**DOI:** 10.1038/s41598-021-94525-6

**Published:** 2021-07-22

**Authors:** Tadashi Itagaki, Hirohiko Motoki, Kyuhachi Otagiri, Keisuke Machida, Takahiro Takeuchi, Masafumi Kanai, Kazuhiro Kimura, Satoko Higuchi, Masatoshi Minamisawa, Hiroshi Kitabayashi, Koichiro Kuwahara

**Affiliations:** 1 Department of Cardiovascular Medicine, Ina Central Hospital, Ina, Japan; 2 Department of Cardiovascular Medicine, Shinshu University School of Medicine, 3-1-1 Asahi, Matsumoto, 390-8621 Japan

**Keywords:** Cardiology, Prognostic markers

## Abstract

The Glasgow Prognostic Score (GPS) has been established as a useful resource to evaluate inflammation and malnutrition and predict prognosis in several cancers. However, its prognostic significance in patients with heart failure (HF) is not well established. To investigate the association between the GPS and mortality in patients with HF, we assessed 870 patients who were 20 years old and more and had been admitted for acute decompensated HF. The GPS ranged from 0 to 2 points as previously reported. Over the 18-month follow-up (follow-up rate, 83.9%), 143 patients died. Increasing GPS was associated with higher HF severity assessed by New York Heart Association functional class and B-type natriuretic peptide (BNP) levels. Kaplan–Meier analysis showed significant associations for mortality and increased GPS. In multivariate analysis, compared to the GPS 0 group, the GPS 2 group was associated with high mortality (hazard ratio 2.92, 95% confidence interval 1.77–4.81, p < 0.001) after adjustment for age, sex, blood pressure, HF history, HF severity, hemoglobin, renal function, sodium, BNP, left ventricular ejection fraction, and anti-HF medications. In conclusion, high GPS was significantly associated with worse prognosis in patients with HF. Inflammation-based assessment by the GPS may enable simple evaluation of HF severity and prognosis.

## Introduction

Heart failure (HF) is predominantly a disease of the elderly^1^. The prevalence of multi-morbidity also increases with age^2^, with elderly patients having five or six comorbidities in addition to HF^3–5^. Frequently coexisting malnutrition also affects the prognosis of patients with HF^6^. Thus, broader interventions beyond HF management are necessary for aged patients, including the treatment of concurrent decompensated chronic conditions, reduction of polypharmacy, minimization of disability, and prescription of physical exercise and nutritional supplementation^7,8^.

Recently, inflammation in addition to neurohormonal activation has been recognized to play an important role in the pathogenesis of both HF with reduced ejection fraction (rEF) and preserved ejection fraction (pEF)^9–14^. Emerging pathophysiological models for HF in the elderly suggest that coexisting proinflammatory cardiovascular and noncardiovascular conditions lead to systemic microvascular endothelial inflammation, global cardiac and skeletal muscle inflammation, and subsequent fibrosis^15,16^. Indeed, increases in inflammatory markers such as C-reactive protein (CRP) have been associated with the severity and prognosis of HF^10,11,17–22^.

The Glasgow Prognostic Score (GPS) is a simple and well-established nutritional and inflammatory assessment tool for patients with malignancy that incorporates serum albumin (Alb) and CRP^23^. Previous studies have validated the GPS as a reliable prognostic indicator in patients with several kinds of cancers^23–27^. We therefore hypothesized that such inflammation-based assessment could also be a prognostic indicator for patients with HF. The aim of this study was to investigate the prognostic ability of the GPS for mortality in patients with HF.

## Results

### Study population

**Figure 1:**
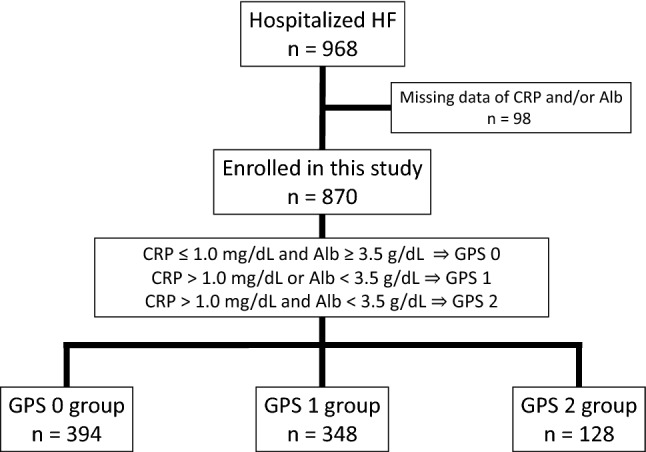
Flow diagram of study recruitment. *Alb* albumin, *CRP* C-reactive protein, *GPS* Glasgow Prognostic Score, *HF* heart failure.

The 870 enrolled patients were categorized into the GPS 0 group (n = 394), GPS 1 group (n = 348), or GPS 2 group (n = 128) based on biochemical measurements (Fig. 1). The baseline patient characteristics stratified by GPS are shown in Table 1. The median [25th, 75th percentile] age of the cohort was 81 [72, 87] years, and 45.4% of patients were female. Patients in the GPS 2 group had significantly lower body mass index, higher heart rate, lower proportion of dyslipidemia and more moderate to severe HF (New York Heart Association [NYHA] functional class III or IV) versus the GPS 1 and 0 groups. The etiology of heart failure and traditional coronary risk factors except for dyslipidemia, including hypertension, and diabetes mellitus, were comparable among the groups. Regarding laboratory data, creatinine, estimated glomerular filtration rate (eGFR), sodium, and hemoglobin A1c values were all similar among the groups. Alb, hemoglobin, and low-density lipoprotein cholesterol tended to decrease, while CRP and B-type natriuretic peptide (BNP) tended to increase, as GPS increased. In echocardiographic examinations, patients in the GPS 0 group had the lowest left ventricular ejection fraction (LVEF). HFpEF (LVEF ≥ 50%) and HFrEF (LVEF < 40%) patients were most prevalent in the GPS 1 group and GPS 0 group, respectively. The use of angiotensin-converting enzyme inhibitor (ACEI) and/or angiotensin-receptor blocker (ARB) and beta-blockers was the most frequent in the GPS 0 group, with no significant differences noted for aldosterone antagonists or loop diuretics.

**Table 1:** Patient characteristics.

	Overall	GPS 0	GPS 1	GPS 2	p-value
	(n = 870)	(n = 394)	(n = 348)	(n = 128)	
Age (years)	81 [72, 87]	77 [67, 84]	84 [77, 88]	84 [78, 88]	< 0.001
Female, n (%)	395 (45.4)	165 (41.9)	182 (52.3)	48 (37.5)	0.002
Body mass index (kg/m^2^)	21.0 [18.9, 23.9]	21.7 [19.5, 24.5]	20.5 [18.5, 23.1]	20.2 [18.6, 23.5]	< 0.001
Heart rate (beats/min)	69 [60, 80]	69 [60, 79]	69 [60, 80]	70 [65, 81]	0.061
Systolic blood pressure (mmHg)	112 [101, 126]	111 [101, 124]	113 [102, 128]	114 [101, 126]	0.30
Hypertension, n (%)	556 (63.9)	240 (60.9)	223 (67.0)	83 (64.8)	0.173
Dyslipidemia, n (%)	229 (26.3)	118 (29.9)	87 (25.0)	24 (18.8)	0.034
Diabetes mellitus, n (%)	256 (29.4)	118 (29.9)	104 (29.9)	34 (26.6)	0.76
NYHA class III or IV, n (%)	170 (19.5)	61 (15.5)	77 (22.1)	32 (25.0)	0.017
Prior HF hospitalization, n (%)	273 (31.4)	133 (33.8)	95 (27.3)	45 (35.2)	0.099
Ischemic etiology, n (%)	229 (26.3)	98 (24.9)	95 (27.3)	36 (28.1)	0.66
Valvular disease, n (%)	267 (30.7)	131 (33.2)	104 (29.9)	32 (25.0)	0.199
Atrial fibrillation, n (%)	448 (51.5)	213 (54.2)	171 (49.1)	64 (50.0)	0.23
**Laboratory data**					
CRP (mg/dL)	0.23 [0.10, 0.71]	0.12 [0.05, 0.26]	0.30 [0.12, 0.60]	2.27 [1.49, 3.84]	< 0.001
Alb (g/dL)	3.4 [3.1, 3.8]	3.8 [3.6, 4.0]	3.2 [3.0, 3.4]	3.0 [2.7, 3.2]	< 0.001
Hemoglobin (g/dL)	12.0 [10.5, 13.7]	13.0 [11.3, 14.6]	11.3 [10.1, 12.8]	11.0 [10.0, 12.4]	< 0.001
Creatinine (mg/dL)	1.08 [0.86, 1.41]	1.09 [0.88, 1.39]	1.07 [0.85, 1.46]	1.10 [0.82, 1.56]	0.87
eGFR (mL/min/1.73 m^2^)	45.0 [33.0, 58.3]	46.1 [34.9, 59.0]	44.8 [32.1, 57.2]	45.1 [31.2, 60.5]	0.27
Sodium (mEq/L)	139 [137, 141]	139 [137, 141]	140 [137, 141]	139 [136, 141]	0.176
LDL cholesterol (mg/dL)	94 [76, 116]	97 [78, 121]	93 [76, 114]	88 [72, 109]	0.027
Hemoglobin A1c (%)	6.0 [5.6, 6.5]	6.0 [5.7, 6.5]	5.9 [5.6, 6.4]	6.0 [5.6, 6.3]	0.079
BNP (pg/mL)	288 [136, 527]	221 [111, 452]	327 [158, 558]	353 [160, 754]	< 0.001
**Echocardiographic data**					
LAD (cm)	4.5 [4.0, 5.0]	4.5 [4.0, 5.0]	4.5 [4.0, 5.0]	4.5 [3.9, 4.9]	0.41
LVDd (cm)	5.0 [4.4, 5.8]	5.3 [4.5, 6.0]	4.9 [4.3, 5.5]	4.8 [4.4, 5.6]	< 0.001
LVDs (cm)	3.6 [2.9, 4.6]	4.0 [3.0, 4.9]	3.4 [2.7, 4.3]	3.3 [2.8, 4.2]	< 0.001
LVEF (%)	49.0 [35.0, 62.0]	44.0 [33.0, 58.0]	54.0 [40.0, 63.0]	53.0 [35.8, 67.2]	< 0.001
LVEF ≥ 50%, n (%)	415 (47.7)	147 (38.0)	202 (59.1)	66 (52.8)	< 0.001
LVEF < 40%, n (%)	286 (32.9)	160 (41.3)	84 (24.6)	42 (33.6)	< 0.001
**Medications**					
ACEI and/or ARB, n (%)	603 (69.3)	292 (74.1)	227 (65.2)	84 (65.6)	0.012
Beta-blockers, n (%)	630 (72.4)	304 (77.2)	251 (72.1)	75 (58.6)	< 0.001
Aldosterone antagonists, n (%)	469 (53.9)	230 (58.4)	180 (51.7)	59 (46.1)	0.070
Loop diuretics, n (%)	733 (84.3)	334 (84.8)	298 (85.6)	101 (78.9)	0.30
**Mortality**					
All-cause death, n (%)	143 (16.4)	35 (8.9)	66 (19.0)	42 (32.8)	< 0.001
Cardiac death, n (%)	86 (9.9)	26 (6.6)	38 (10.9)	22 (17.2)	0.002
Noncardiac death, n (%)	54 (6.2)	8 (2.0)	27 (7.8)	19 (14.8)	< 0.001
Unknown death, n (%)	3 (0.3)	1 (0.2)	1 (0.3)	1 (0.8)	

Continuous variables are summarized as the median and interquartile range. Comparisons between patient groups were performed using the Kruskal–Wallis test for continuous variables and by means of contingency table analysis and Fisher’s exact test for categorical variables. *ACEI* angiotensin-converting enzyme inhibitor, *Alb* albumin, *ARB* angiotensin-receptor blocker, *BNP* B-type natriuretic peptide, *CRP* C-reactive protein, *Dd* end-diastolic diameter, *Ds* end-systolic diameter, *EF* ejection fraction, *eGFR* estimated glomerular filtration rate, *GPS* Glasgow Prognostic Score, *HF* heart failure, *LAD* left atrial dimension, *LDL* low-density lipoprotein, *LV* left ventricular, *NYHA* New York Heart Association.

### Relationship between GPS and clinical characteristics

Age, BNP, and LVEF correlated positively with GPS (r = 0.300, p < 0.001; r = 0.182, p < 0.001; and r = 0.170, p < 0.001, respectively), whereas hemoglobin correlated negatively with GPS (r = − 0.322, p < 0.001). There were no significant correlations between GPS and systolic blood pressure, eGFR, or sodium (Table 2).

**Table 2:** Univariate correlations between the GPS and baseline indices.

Variable	Spearman’s r	p-value
Age (years)	0.300	< 0.001
Systolic blood pressure (mmHg)	0.050	0.139
Hemoglobin (g/dL)	− 0.332	< 0.001
eGFR (mL/min/1.73 m^2^)	− 0.049	0.152
Sodium (mEq/L)	− 0.005	0.90
BNP (pg/mL)	0.182	< 0.001
LVEF (%)	0.170	< 0.001

Correlations between GPS and clinical and laboratory indices were evaluated by Spearman’s rank test. *BNP* B-type natriuretic peptide, *eGFR* estimated glomerular filtration rate, *GPS* Glasgow Prognostic Score, *LVEF* left ventricular ejection fraction.

### Association of GPS with mortality

**Figure 2:**
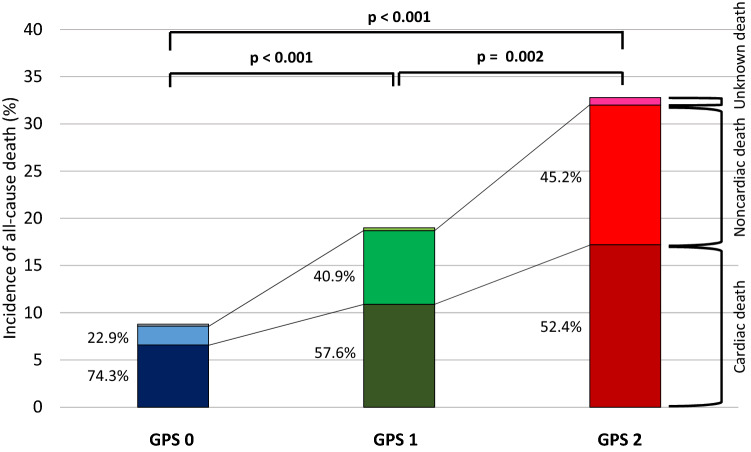
Incidence of all-cause death stratified by GPS and cause of deaths. Higher incidence of all-cause, cardiac and noncardiac mortality was observed with increasing GPS. *GPS* Glasgow Prognostic Score.

**Figure 3:**
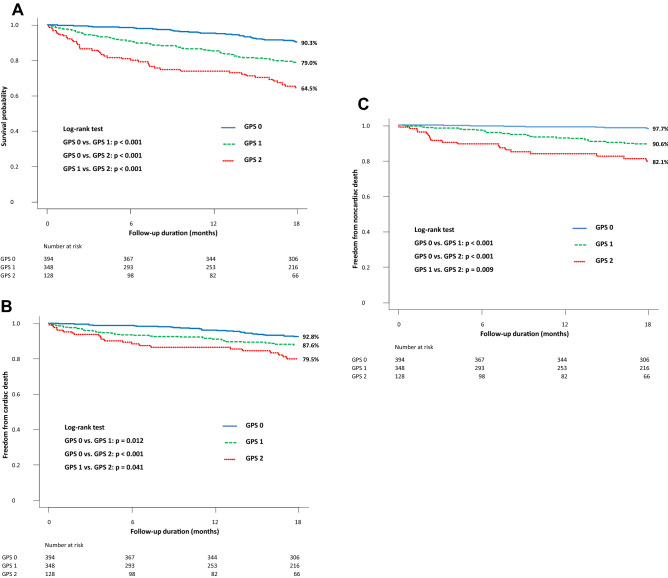
Kaplan–Meier survival curve of all-cause, cardiac and noncardiac mortality stratified by GPS. (**A**) Higher GPS was associated with worse prognosis in the overall cohort. With increasing the GPS, (**B**) cardiac mortality and (**C**) noncardiac mortality increased. *GPS* Glasgow Prognostic Score.

Of the 870 patients, 143 (16.4%) died during the 18-month (548-day) follow-up period. Follow-up rates at 6 months, 12 months and 18 months were 94.0%, 89.2% and 83.9%, respectively. Incidence of all-cause mortality increased significantly with increasing GPS (GPS 0 vs. 1 vs. 2: 8.9% vs. 19.0% vs. 32.8%; GPS 0 vs. 1 or 2: both p < 0.001 and GPS 1 vs. 2: p = 0.002) (Table 1, Fig. 2). Moreover, there were significant differences in cardiac death (p = 0.002) and noncardiac death (p < 0.001) among the 3 groups (Table 1). Cardiac death accounted for 74.3%, 57.6% and 52.4% of all-cause death in the GPS 0, 1 and 2 groups, respectively. On the other hand, noncardiac death accounted for 22.9%, 40.9% and 45.2% of all-cause death in the GPS 0, 1, and 2 groups, respectively (Fig. 2). In Kaplan–Meier survival analysis, patients in the GPS 2 group had a significantly lower survival probability than did those in the GPS 1 group (64.5% vs. 79.0%, log-rank p < 0.001) and the GPS 0 group (90.3%, p < 0.001). GPS 1 patients also had a significantly lower survival probability than did GPS 0 patients (p < 0.001) (Fig. 3A). As with all-cause mortality, cardiac mortality and noncardiac mortality increased significantly with increasing GPS (Fig. 3B,C). Multivariate Cox proportional hazards analysis adjusted for age, sex, systolic blood pressure, NYHA functional class, prior HF hospitalization, hemoglobin, eGFR, sodium, BNP, LVEF, and the use of ACEI and/or ARB, beta-blockers, or aldosterone antagonists revealed that the hazard ratio for mortality in GPS 1 and 2 patients relative to GPS 0 patients were 1.53 (95% confidence interval [CI] 0.96–2.43, p = 0.071) and 2.92 (95% CI 1.77–4.81, p < 0.001), respectively (Table 3). C-statistics for all-cause-mortality were greater in baseline model plus GPS (0.754, p = 0.046) than baseline model alone (0.733). The net reclassification improvement (NRI) and integrated discrimination improvement (IDI) for all-cause mortality also significantly increased after addicting GPS to baseline model (0.249, p = 0.006; 0.029, p < 0.001, respectively) (Table 4).

**Table 3:** Univariate and multivariate cox proportional hazards analysis to identify prognostic ability of the GPS.

	Univariate analysis		Multivariate analysis	
	HR (95% CI)	p-value	HR (95% CI)	p-value
GPS 0	Reference		Reference	
GPS 1	2.41 (1.60–3.63)	< 0.001	1.53 (0.96–2.43)	0.071
GPS 2	4.62 (2.95–7.24)	< 0.001	2.92 (1.77–4.81)	< 0.001
Age (years)	1.07 (1.05–1.09)	< 0.001	1.06 (1.04–1.09)	< 0.001
Female, n (%)	0.86 (0.62–1.21)	0.39	0.67 (0.46–0.99)	0.042
Systolic blood pressure (mmHg)	0.99 (0.981–0.999)	0.032	0.99 (0.981–1.001)	0.064
NYHA class III or IV, n (%)	2.13 (1.50–3.03)	< 0.001	1.33 (0.89–1.98)	0.166
Prior HF hospitalization, n (%)	1.83 (1.32–2.55)	< 0.001	1.29 (0.90–1.87)	0.168
Hemoglobin (g/dL)	0.79 (0.72–0.85)	< 0.001	0.89 (0.81–0.99)	0.030
eGFR (mL/min/1.73 m^2^)	0.97 (0.96–0.98)	< 0.001	0.98 (0.97–0.99)	0.002
Sodium (mEq/L)	0.96 (0.916–1.001)	0.056	0.98 (0.94–1.03)	0.41
BNP (pg/mL)	1.00 (1.000–1.001)	< 0.001	1.00 (1.000–1.001)	0.052
LVEF (%)	0.99 (0.983–1.004)	0.191	0.99 (0.977–1.002)	0.103
ACEI and/or ARB, n (%)	0.83 (0.59–1.18)	0.30	1.04 (0.72–1.51)	0.82
Beta-blockers, n (%)	0.68 (0.48–0.96)	0.029	0.90 (0.61–1.34)	0.62
Aldosterone antagonists, n (%)	0.89 (0.63–1.22)	0.44	1.04 (0.72–1.50)	0.83

Data presented are hazard ratios and 95% confidence intervals. *ACEI* angiotensin-converting enzyme inhibitor, *ARB* angiotensin-receptor blocker, *BNP* B-type natriuretic peptide, *CI* confidence interval, *eGFR* estimated glomerular filtration rate, *GPS* Glasgow Prognostic Score, *HF* heart failure, *HR* hazard ratio, *LVEF* left ventricular ejection fraction, *NYHA* New York Heart Association.

**Table 4:** Discrimination of each predictive model for all-cause mortality using C-statistics, NRI and IDI.

Predictive models	C-statistics (95% CI)	p value	NRI	p value	IDI	p value
Baseline model	0.733 (0.687–0.778)	Reference	Reference		Reference	
+ GPS	0.754 (0.710–0.798)	0.046	0.249	0.006	0.029	< 0.001

Baseline model included age, sex, hypertension, diabetes mellitus, atrial fibrillation, New York Heart Association functional class. *CI* confidence interval, *GPS* Glasgow Prognostic Score, *IDI* integrated discrimination improvement, *NRI* net reclassification improvement.

### Subgroup analysis

**Figure 4:**
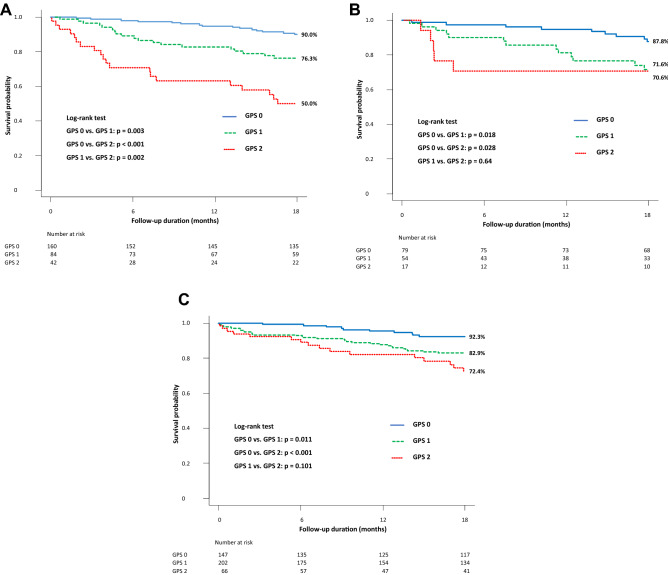
Kaplan–Meier survival curve of patients by HF subtype. (**A**) Survival probability decreased significantly with increasing GPS in HFrEF patients. (**B**) HFmrEF patients with GPS 0 had significantly higher survival probability than did those with GPS 1 or 2. (**C**) Although HFpEF patients with GPS 0 had significantly higher survival probability than did those with GPS 1, no other remarkable differences were seen. *GPS* Glasgow Prognostic Score, *HFmrEF* heart failure with mid-range ejection fraction, *HFpEF* heart failure with preserved ejection fraction, *HFrEF* heart failure with reduced ejection fraction.

To evaluate the association of GPS with mortality according to HF subtype, the cohort was divided into the HFpEF (LVEF ≥ 50%), HF with mid-range EF (HFmrEF) (LVEF ≥ 40% and < 50%), and HFrEF (LVEF < 40%) groups. In HFrEF patients, incidence of all-cause death increased significantly with increasing GPS (GPS 0 vs. 1 vs. 2: 9.4% vs. 22.6% vs. 47.6%; GPS 0 vs. 1 or 2: p = 0.006 or p < 0.001 and GPS 1 vs. 2: p = 0.007) (Fig. S1A online). Although there was no significant difference between three groups, mortality tended to increase with GPS in the HFmrEF subgroup (GPS 0 vs. 1 or 2: 11.4% vs 24.1% or 29.4%, p = 0.061 or p = 0.121; GPS 1 vs. 2: p = 0.75) (Fig. S1B online). The GPS 0 group had lower mortality rate than the GPS 1 group (6.8% vs. 15.3%, p = 0.018) and the GPS 2 group (vs. 24.2%, p = 0.001). There was no significant difference between the GPS 1 and 2 group (p = 0.134) (Fig. S1C online). In Kaplan–Meier analysis, survival probability decreased significantly with increasing GPS in HFrEF patients (GPS 0 vs. 1 vs. 2: 90.0% vs. 76.3% vs. 50.0%; log-rank test: GPS 0 vs. 1 or 2: p = 0.003 or p < 0.001 and GPS 1 vs. 2: p = 0.002) (Fig. 4A). In HFmrEF patients, those with GPS 0 had a higher survival probability than did those with GPS 1 or 2 (87.8% vs. 71.6% or 70.6%; p = 0.018 or p = 0.028). There was no significant difference in mortality between patients with GPS 1 and 2 (p = 0.64) (Fig. 4B). Similarly, in HFpEF patients, those with GPS 0 had a higher survival probability than did those with GPS 1 or 2 (92.3% vs. 82.9% or 72.4%; p = 0.011 or p < 0.001). There was no significant difference in mortality between patients with GPS 1 and 2 (p = 0.101) (Fig. 4C).

## Discussion

This study demonstrated the prognostic significance of the GPS for predicting mortality in patients with HF. Specifically, it showed that: (1) patients with higher GPS had significantly higher severity of HF assessed by NYHA functional class and BNP levels than did patients with lower GPS, (2) age, BNP, and LVEF all correlated positively with GPS, (3) high GPS was significantly associated with a worse prognosis, (4) the GPS improve the predictive accuracy of all-cause mortality compared to other predictive model that did not require any form of laboratory testing, (5) both cardiac mortality and noncardiac mortality increased with increasing GPS, (6) better prognosis were observed in the GPS 0 group in all HF subtype groups and (7) significant associations between GPS and mortality were more evident in patients with HFrEF.

The GPS has been identified as a reliable predictor of mortality in several cancers, including non-small-cell lung cancer, gastro-esophageal cancer, and hepatocellular carcinoma^23–25^. Chronic activation of the systemic inflammatory response induces cancer progression. Inflammation leads to the accumulation of regulatory T lymphocytes and chemokines as well as the stimulation of cytokines, which produce CRP, increase neutrophils, and induce an inappropriate immune response. As a result, tumor angiogenesis, invasion, and metastasis are promoted^25^. Moreover, systemic inflammation causes malnutrition leading to decreased body weight, fatigue, loss of lean mass, declines in activities of daily living, and poor survival^24^. It is also considered that the progression of sarcopenia and malnutrition due to systemic inflammation may contribute to poor outcomes in patients with HF. Indeed, our study showed that high GPS was significantly associated with a worse prognosis in patients with HF. These are consistent with a previous report by Namiuchi et al., which found that higher GPS was an independent risk factor for higher mortality among admitted patients with acute decompensated HF^28^. However, the present study analyzed the GPS for risk-stratification in patients with HF in an older and larger cohort than the above report^28^. A few studies have reported the prognostic value of the GPS in HF according to HF subtype. Cho et al. recently demonstrated the utility of the modified GPS for predicting survival in HFrEF patients^29^ and Bolat et al. reported the modified GPS as the novel predictor of clinical outcome including all-cause mortality in HFpEF patients^30^. Our findings supported their studies^29,30^. In the present study, as GPS increased, mortality had tendency to increase in patients with HFrEF and HFpEF. In particular, the tendency was evident and significant in patients with HFrEF. Additionally, in all subtype of HF including HFmrEF, low GPS was suggested to associate with better prognosis. Further assessment of nutrition and inflammation is needed for a broader HF population age spectrum and more analyses according to HF subtype.

In patients with HF, hypoalbuminemia is caused by multiple factors, including anorexia, malabsorption, high energy demand, and cytokine-induced increases in metabolic rate^31^. Hypoalbuminemia may exacerbate HF severity by promoting peripheral and pulmonary congestion and cause increased oxidative stress and inflammation to create a vicious cycle of worsening inflammation, HF, and hypoalbuminemia^31^. HF also provokes inflammation by wall stress and the release of cardiac cytokines and other inflammatory mediators from the heart and other organs. Cytokines evoke myocardial remodeling and pump failure, resulting in the progression of HF^14^.

This study demonstrated that the GPS was useful for estimating the prognosis of patients with HF. Additionally, the GPS was associated with the severity of HF because the number of patients with NYHA functional class III or IV and levels of BNP increased with increasing GPS and BNP correlated positively with GPS, which indices are recognized to reflect severity of HF^5,32^. The GPS can predict mortality using only two routinely measured parameters, CRP and Alb, both of which can be easily obtained in clinical practice. Several nutritional indicators, such as the GNRI, PNI, and CONUT scores, as well as inflammatory markers including CRP, have been reported to individually associate with HF prognosis and severity^6,10,11,17–22^. Prognosticators that consist of both nutritional and inflammatory markers would therefore appear most useful for patients with HF. Since the GPS reflects systemic inflammation (elevated CRP) and malnutrition (hypoalbuminemia), it may be considered appropriate to reflect the severity of HF. Moreover, nutritional assessment alone isn’t enough to evaluate severity of inflammation and treat it, which is one of the causes of malnutrition. Similarly, inflammation assessment alone isn’t enough to evaluate how much inflammation affects nutritional status. Assessment of combining nutritional status and inflammation allow not only nutritional intervention but also approach chronic inflammatory diseases in the background of malnutrition. Finally, the improvement of patient GPS by the early management of nutrition and inflammation may therefore improve also prognosis.

In this study, all-cause mortality was chosen as primary endpoint. Along with the aging society, the number of elderly HF patients is increasing^33,34^. Although the most common cause of death in patients with HF is cardiac death regardless of HF subtype^35,36^, it has been reported that the proportion of noncardiac death in elderly HF patients is higher than in younger HF patients^37^. Thus, it may be important to predict all-cause mortality as well as cardiac mortality, especially in elderly HF patients. Furthermore, because of increasing frailty and progression of HF in elderly patients, palliative and end-of-life care has been needed for them. However, it is often difficult to determine when to introduce palliative care for elderly HF patients, because of their clinical course before death which differ from cancer patients. Patients with HF have repeated acute exacerbations and diminished their physical function. Each exacerbation can lead to death, but usually symptoms at that time improve with treatment. Whereas, their conditions rapidly deteriorate before they die^38,39^. Hence, it is also difficult to recognize that they are at the end-of-life^40^. To predict not cardiac mortality but all-cause mortality by using the GPS may help introducing palliative care for elderly HF patients at the appropriate time.

This study had several limitations. First, the sample size was relatively limited; fewer significant differences in the subgroup analyses may have been caused by underpowered statistics. Second, since we evaluated a cohort of admitted patients with HF, our results might not be generalizable to patients with stable HF. Third, our analysis was based on a single data point taken at discharge, with no further testing during follow-up. Therefore, the impact of GPS improvement on mortality is unknown and requires further study. Fourth, the GPS is associated with acute infection or chronic inflammation such as cancer and collagen disease. There were probably no patients with acute infection in this study, because their Alb and CRP were measured at discharge, and at that time they had no clinical symptoms and physical findings of infection. However, we could not exclude patients with cancer or collagen disease. Finally, because mortality data in this study was obtained from participating institutes instead of a governmental database, our data did not accurately assess mortality in our geographic region. Despite these limitations, however, inflammation-based assessment using the GPS appears useful for the risk stratification of mortality in patients with HF.

In conclusion, high GPS was associated with worse prognosis in patients with HF. Combined assessment of inflammation and nutrition may improve the evaluation of HF severity and prognosis and assist in patient treatment.

## Methods

### Study design and patient population

The present multi-center, prospective, observational study was conducted in Nagano Prefecture, Japan. The inclusion criteria were 20 years of age or more and admission for acute decompensated HF. Patients with acute coronary syndrome (ACS) were excluded. After providing informed consent, subjects were enrolled between July 2014 and December 2018 during a compensated HF state before discharge. We recorded medical history, HF etiology, comorbidities, socio-economic background, medications, and examination findings at discharge that included electrocardiogram (ECG), echocardiography, and blood test results when HF compensated and causes of HF such as arrythmia, anemia and infection were cured. HF and ACS were diagnosed by attending physicians based on symptoms, ECGs, echocardiography, laboratory data, chest X-rays, and coronary angiograms according to the Framingham criteria^41^ and ACC/AHA guidelines^42^, respectively. Hypertension was defined as systolic blood pressure ≥ 140 mmHg, diastolic blood pressure ≥ 90 mmHg, or ongoing therapy of for hypertension. Dyslipidemia was defined as a low-density lipoprotein cholesterol concentration ≥ 140 mg/dL, a high-density lipoprotein cholesterol concentration < 40 mg/dL, a triglyceride concentration ≥ 150 mg/dL, a previous diagnosis of dyslipidemia or current treatment for lipid lowering agents. Diabetes mellitus was defined as a hemoglobin A1c level ≥ 6.5%, fasting plasma glucose ≥ 126 mg/dL, 2-h plasma glucose level after a 75 g oral glucose tolerance test ≥ 200 mg/dL, casual plasma glucose ≥ 200 mg/dL, a previous diagnosis of diabetes mellitus or treatment with oral hypoglycemic agents or insulin injection. LVEF was measured by the biplane modified Simpson’s method, which recommended by the American Society of Echocardiography^43^. Details of the GPS have been described previously. Patients with both elevated CRP (> 1.0 mg/dL) and hypoalbuminemia (< 3.5 g/dL) were allocated a score of 2. Patients in whom only one of these biochemical abnormalities was present were allocated a score of 1. Patients in whom neither of these abnormalities existed were allocated a score of 0 (Fig. 1)^23,44^. Enrolled patients were categorized into the GPS 0, 1, or 2 group based on their GPS at discharge. The study protocol was approved by the Shinshu University Institutional Review Board (approval number: 4237). Informed consent was obtained from all patients. This study was performed in accordance with the Declaration of Helsinki, and is registered with the University Hospital Medical Information Network (UMIN 000024470).

### Endpoint and follow-up

The study endpoint was all-cause mortality. Relationships between mortality rates and GPS were statistically assessed. We followed patients prospectively and collected relevant clinical data at the time of scheduled follow-ups by telephone. Survival status was confirmed by chart review.

### Statistical analysis

Continuous variables are summarized as the mean ± standard deviation if normally distributed and as the median and interquartile range otherwise. Normality was assessed by the Shapiro–Wilk W test. Categorical variables are presented as numbers and percentages. Comparisons between patient groups were performed using the Kruskal–Wallis test for continuous variables and by means of contingency table analysis and Fisher’s exact test for categorical variables. Correlations between GPS and clinical and laboratory indices were evaluated by Spearman’s rank test. Kaplan–Meier survival curves were calculated from baseline to the time of death for comparisons using the log-rank test. Cox proportional hazards regression analysis was conducted to identify the prognostic ability of the GPS. The multivariate analysis model was adjusted for age, sex, systolic blood pressure, NYHA functional class, prior HF hospitalization, hemoglobin, eGFR, sodium, BNP, LVEF, and the use of ACEi and/or ARB, beta-blockers, or aldosterone antagonists. These covariates were selected in advance which are recognized as prognostic factors in HF^45–47^. To evaluate whether the accuracy of predicting all-cause mortality would improve after the addition of the GPS into a baseline model with established risk factors, including age, sex, hypertension, diabetes mellitus, atrial fibrillation, NYHA functional class, C-statistics, NRI and IDI were calculated. The C-statistics is defined as the area under receiver-operating characteristic curves. A p-value of < 0.05 was considered statistically significant. All statistical analyses were performed with R (The R Foundation for Statistical Computing, Vienna, Austria) and EZR (Saitama Medical Center, Jichi Medical University, Saitama, Japan), which is a graphical user interface for R^48^.

### IRB information

This study was approved by the Ethics Committee of Shinshu University Hospital (approval number: 4237) and is registered with the University Hospital Medical Information Network (UMIN 000024470).

## Supplementary Information

Supplementary Figure S1A.

Supplementary Figure S1B.

Supplementary Figure S1C.

Supplementary Figure Caption.

## Data Availability

The deidentified participant data will be shared on a request basis. Please contact the corresponding author to request data sharing. All data, including patients’ clinical characteristics, outcome data, related documents on study protocol, and statistical analysis plan, will be available by digital media to medical doctors for all types of analyses for a period of 1 year following publication of the study.
