# Supplementary figures and images for: Inflammation-based assessment for the risk stratification of mortality in patients with heart failure
Source: Sci Rep. 2021 Jul 22;11:14989. doi: 10.1038/s41598-021-94525-6 (PMC8298574; doi:10.1038/s41598-021-94525-6)

## Slide 1
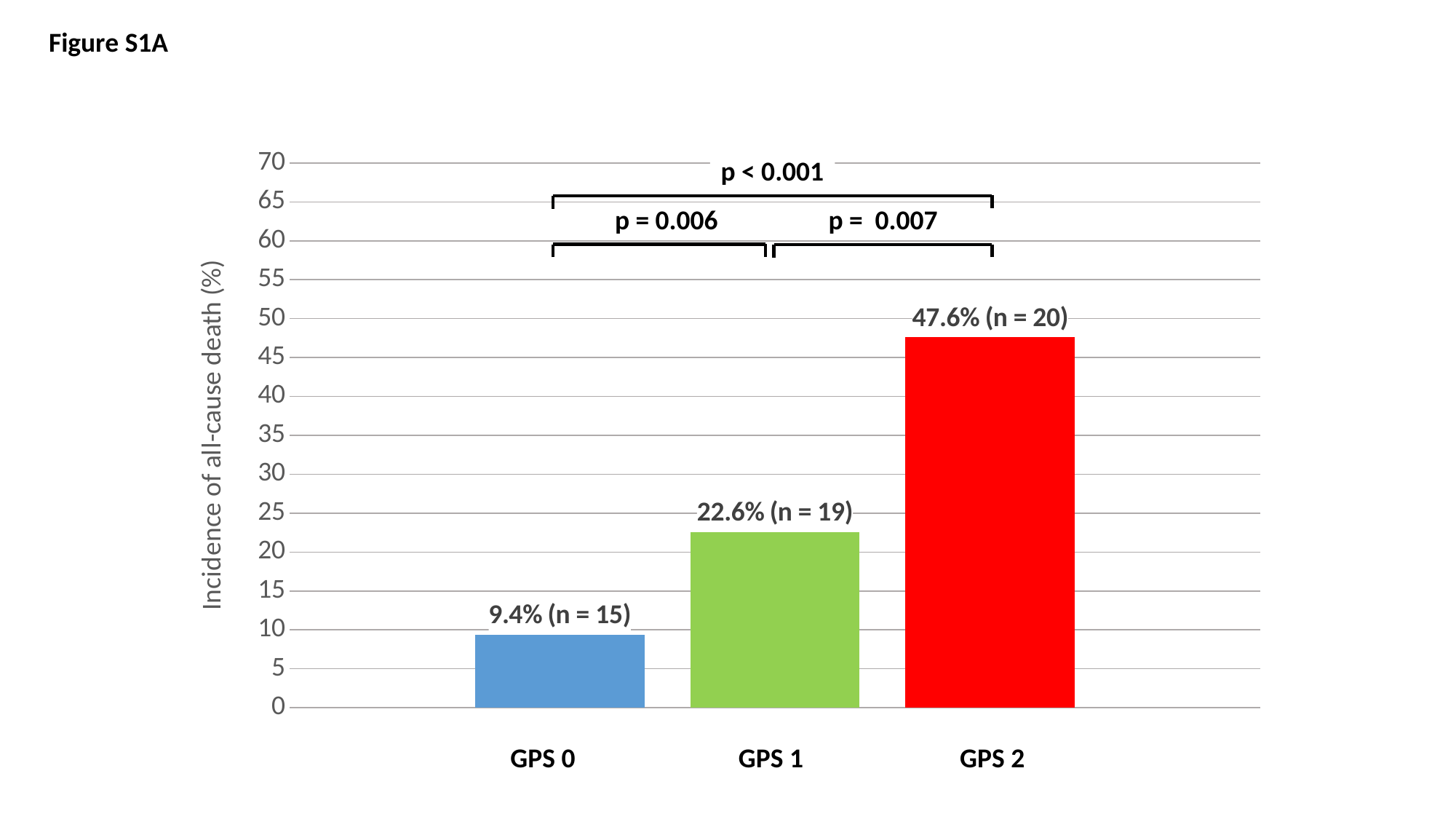

Figure S1A
### Chart
| Category | GPS 0 | GPS 1 | GPS 2 |
|---|---|---|---|
| Mortality | 9.4 | 22.6 | 47.6 |GPS 0
GPS 1
GPS 2
p < 0.001
p = 0.006
p = 0.007

Supplement: Supplementary file 1 — Supplementary Figure S1A. [file 41598_2021_94525_MOESM1_ESM.pptx]

## Slide 1
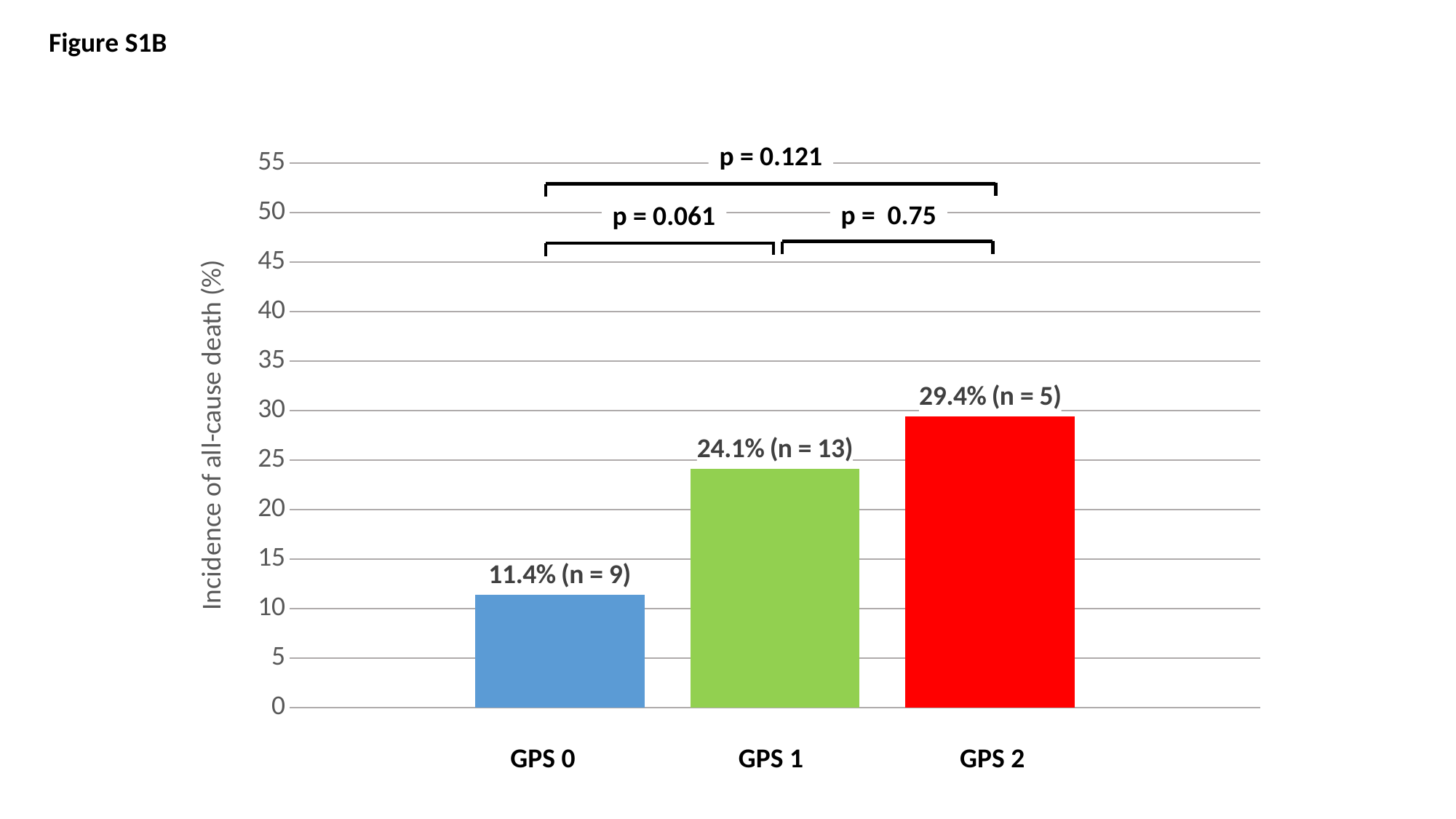

Figure S1B
p = 0.121
### Chart
| Category | GPS 0 | GPS 1 | GPS 2 |
|---|---|---|---|
| Mortality | 11.4 | 24.1 | 29.4 |GPS 0
GPS 1
GPS 2
p = 0.75
p = 0.061

Supplement: Supplementary file 2 — Supplementary Figure S1B. [file 41598_2021_94525_MOESM2_ESM.pptx]

## Slide 1
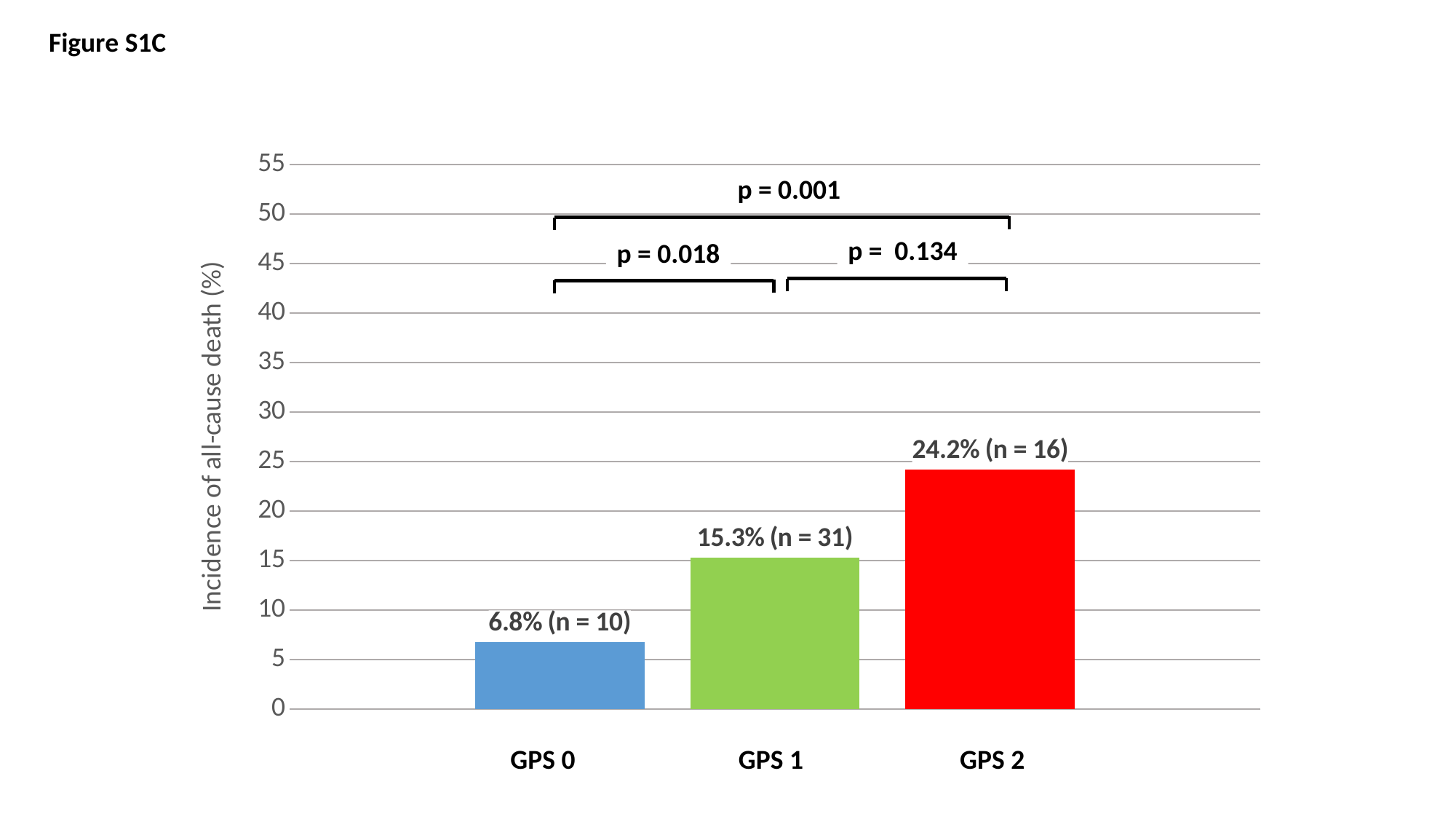

Figure S1C
### Chart
| Category | 6.8 | 14.9 | 24.2 |
|---|---|---|---|
| Mortality | 6.8 | 15.3 | 24.2 |GPS 0
GPS 1
GPS 2
p = 0.001
p = 0.134
p = 0.018

Supplement: Supplementary file 3 — Supplementary Figure S1C. [file 41598_2021_94525_MOESM3_ESM.pptx]
