# Supplementary material for: Inflammation-based assessment for the risk stratification of mortality in patients with heart failure
Source: Sci Rep. 2021 Jul 22;11:14989. doi: 10.1038/s41598-021-94525-6 (PMC8298574; doi:10.1038/s41598-021-94525-6)
Supplement: Supplementary file 4 — Supplementary Figure Caption. [file 41598_2021_94525_MOESM4_ESM.docx]

**Figure S1** **Incidence of mortality in HFrEF, HFmrEF, and HFpEF subgroups**

(A) Association of mortality rate with higher GPS was evident in HFrEF patients. (B) Mortality tended to increase with GPS in the HFmrEF subgroup. (C) No significant difference in mortality among GPS subgroups in HFpEF patients.

GPS, Glasgow Prognostic Score; HFmrEF, heart failure with mid-range ejection fraction; HFpEF, heart failure with preserved ejection fraction; HFrEF, heart failure with reduced ejection fraction
